# WGS of intrauterine *E. coli* from cows with early postpartum uterine infection reveals a non-uterine specific genotype and virulence factors

**DOI:** 10.1128/mbio.01027-24

**Published:** 2024-05-14

**Authors:** Adriana Garzon, Carl Basbas, Cory Schlesener, Noelia Silva-del-Rio, Betsy M. Karle, Fabio S. Lima, Bart C. Weimer, Richard V. Pereira

**Affiliations:** 1 Department of Population Health and Reproduction, School of Veterinary Medicine, University of California, Davis, California, USA; 2 Department of Population Health and Reproduction, 100K Pathogen Genome Project, School of Veterinary Medicine, University of California, Davis, California, USA; 3 Veterinary Medicine Teaching and Research Center, School of Veterinary Medicine, University of California, Tulare, California, USA; 4 Cooperative Extension, Division of Agriculture and Natural Resources, University of California, Orland, California, USA; Cornell University, Ithaca, New York, USA

**Keywords:** whole-genome sequencing, cattle, mutation, antibiotic resistance, uterine infection

## Abstract

*Escherichia coli* has been attributed to playing a major role in a cascade of events that affect the prevalence and severity of uterine disease in cattle. The objectives of this project were to (i) define the association between the prevalence of specific antimicrobial resistance and virulence factor genes in *E. coli* with the clinical status related to uterine infection, (ii) identify the genetic relationship between *E. coli* isolates from cows with diarrhea, with mastitis, and with and without metritis, and (iii) determine the association between the phenotypic and genotypic antimicrobial resistance identified on the *E. coli* isolated from postpartum cattle. Bacterial isolates (*n* = 148) were obtained from a larger cross-sectional study. Cows were categorized into one of three clinical groups before enrollment: metritis, cows with purulent discharge, and control cows. For genomic comparison, public genomes (*n* = 130) from cows with diarrhea, mastitis, and metritis were included in a genome-wide association study, to evaluate differences between the drug classes or the virulence factor category among clinical groups. A distinct *E. coli* genotype associated with metritis could not be identified. Instead, a high genetic diversity among the isolates from uterine sources was present. A virulence factor previously associated with metritis (*fim*H) using PCR was not associated with metritis. There was moderate accuracy for whole-genome sequencing to predict phenotypic resistance, which varied depending on the antimicrobial tested. Findings from this study contradict the traditional pathotype classification and the unique intrauterine *E. coli* genotype associated with metritis in dairy cows.

**IMPORTANCE:**

Metritis is a common infectious disease in dairy cattle and the second most common reason for treating a cow with antimicrobials. The pathophysiology of the disease is complex and is not completely understood. Specific endometrial pathogenic *Escherichia coli* have been reported to be adapted to the endometrium and sometimes lead to uterine disease. Unfortunately, the specific genomic details of the endometrial-adapted isolates have not been investigated using enough genomes to represent the genomic diversity of this organism to identify specific virulence genes that are consistently associated with disease development and severity. Results from this study provide key microbial ecological advances by elucidating and challenging accepted concepts for the role of Intrauterine *E. coli* in metritis in dairy cattle, especially contradicting the existence of a unique intrauterine *E. coli* genotype associated with metritis in dairy cows, which was not found in our study.

## INTRODUCTION

Metritis is a common infectious disease in dairy cattle and the second most common reason for treating a cow with antibiotics (1). Metritis typically occurs within 21 days postpartum, characterized by an enlarged uterus and fetid, watery red-brown uterine discharge with systemic signs of illness (2). Metritis also has an important effect on herd profitability (3). The cost of a metritis case in cows treated with ampicillin and with ceftiofur has been estimated to average $344 and $410, respectively (4). However, the clinical and reproductive beneficial influence of antibiotic treatment has yielded inconsistent results (5). The variability in treatment success may be explained in part by the lack of a gold standard for disease diagnosis, the self-cure rate associated with the disease, and the variability of including control groups that hinder the comparison among studies.

The pathophysiology of the disease is complex with limited studies that generate information to allow causal inferences about the disease (6). Part of this challenge includes a lack of consensus on the criteria and terminology used for clinical disease diagnosis in research studies, as recently demonstrated in a scoping review (ScR) (7). Although inconsistencies in the clinical definition of metritis exist, the aforementioned ScR identified that most studies defined metritis as a fetid, red-brown watery discharge with or without systemic signs of disease based on the work of Sheldon et al. (2).

The reproductive tract in cows harbors a diverse microbiome (8–10), and recent advancements in next-generation sequencing have demonstrated that the uterus has an established microbiome even before parturition and does not exclusively result from microbial exposure during calving (11–13). Several bacteria have been isolated from metritic cows and are suspected as causal agents of metritis, including *Escherichia coli*, *Trueperella pyogenes*, *Fusobacterium necrophorum*, and *Prevotella* spp. (14, 15). Descriptive studies using 16S rRNA sequencing revealed a higher abundance of *Bacteroides*, *Porphyromonas*, and *Fusobacterium* species in cows with metritis when compared with healthy cows, while *Escherichia coli* bacteria were found to be more abundant in healthy animals (11, 16, 17). *In vivo* models of uterine infections have been successfully developed using pathogenic *T. pyogenes* alone (18), in combination with *E. coli* (19) or as a pathogenic cocktail of *T. pyogenes*, *E. coli*, and *F. necrophorum* (20), yet the disease causality has not been demonstrated with other bacterial species.

Based on current data for *Escherichia coli* in metritic cows, these bacteria have been proposed to play a major role in a cascade of events that affect the prevalence, severity, and persistence of uterine disease in cattle (6). Specific endometrial pathogenic *Escherichia coli* (EnPEC) have been described as adapted to the endometrium, allowing them to colonize this environment and develop uterine disease (21), as well as increasing the susceptibility of the endometrium to infection with *T*. *pyogenes* (22).

Most of the data for intrauterine *E. coli* from cows with metritis are either based on culture-based methods or using PCR, which has a limited screening to a few preselected known virulence genes, found specific virulence factors and antimicrobial resistance genes associated to uterine disease occurrence (23–26). Previous genomic comparisons of intrauterine *E. coli* from metritic cows from whole-genome sequencing (WGS) used low-depth sequencing from very few isolates that do not represent the genomic diversity of the organism (27). The high genetic diversity and genomic plasticity of *E. coli* are well recognized (28) and must be considered with examining genomic features for causality in disease. Therefore, genome-wide association studies including genomes from control animals and different infection sources and at a sufficient genome scale are required to identify specific adaptive traits in multi-factorial diseases *in vivo*.

Extended-spectrum beta-lactamase (ESBL) confers resistance to a wide range of beta-lactam antimicrobials, including third-generation cephalosporins, such as ceftiofur, the most used antimicrobial for metritis treatment. Livestock have been recognized as reservoirs for ESBL-producing *E. coli* and therefore have been assumed to represent a source for disseminating and spreading this important AMR genetic element to human populations (29, 30). Molecular approaches to characterize ESBL genes in dairy cattle and compare the prevalence between infectious diseases that most frequently received antimicrobial treatment (1, 31) could guide future efforts for treatment success and AMR surveillance programs.

We hypothesized that *E. coli* isolated from postpartum dairy cows would have unique distinctive genomic characteristics, including virulence genes when compared with isolates from cows without metritis. To test this hypothesis three objectives were undertaken: (i) identify the prevalence of VF and AMR genes in *E. coli* isolated from postpartum dairy cattle from 25 commercial dairy farms in California, (ii) determine the specific genes associated with clinical disease via population comparative microbial genomics, and (iii) determine the association between the antimicrobial phenotypic resistance and the antimicrobial resistance genes identified on the *E. coli* isolated from postpartum cattle.

## RESULTS

An isolate set from this study of 148 *E. coli* WGS was used for population comparisons. Out of the 148 genomes, 54 were retrieved from cows categorized based on the vaginal discharge (VD) as cows with metritis (MET), 45 from cows with purulent discharge (PUS), and 49 from cows with normal discharge (CTL) (Table 1). The genomes ranged from 4,526,522 to 6,358,509 bp assembled per genome. An additional 130 genomes were retrieved from NCBI, but 8 were excluded due to poor quality, and 2 genomes were excluded due to contamination, resulting in a total of 120 additional genomes being included in the population analysis. The genomes ranged from 4,580,125 to 5,481,723 bp assembled per genome (in Tables S1 and S2).

**TABLE 1:** Descriptive information for 148 postpartum dairy cows from which *E. coli* uterine isolates were retrieved in California dairy farms

Variable	Metritis	Pus	Control
*n* of cows	54	45	49
DIM^*a*^	8 (3–21)	10 (3–21)	9.5 (3–21)
Lactation	2 (1–10)	3 (1–6)	2 (1–6)
Farms sampled	23	21	21

^*a*^ DIM: days in milk.

### Comparative population genomics

**Fig 1:**
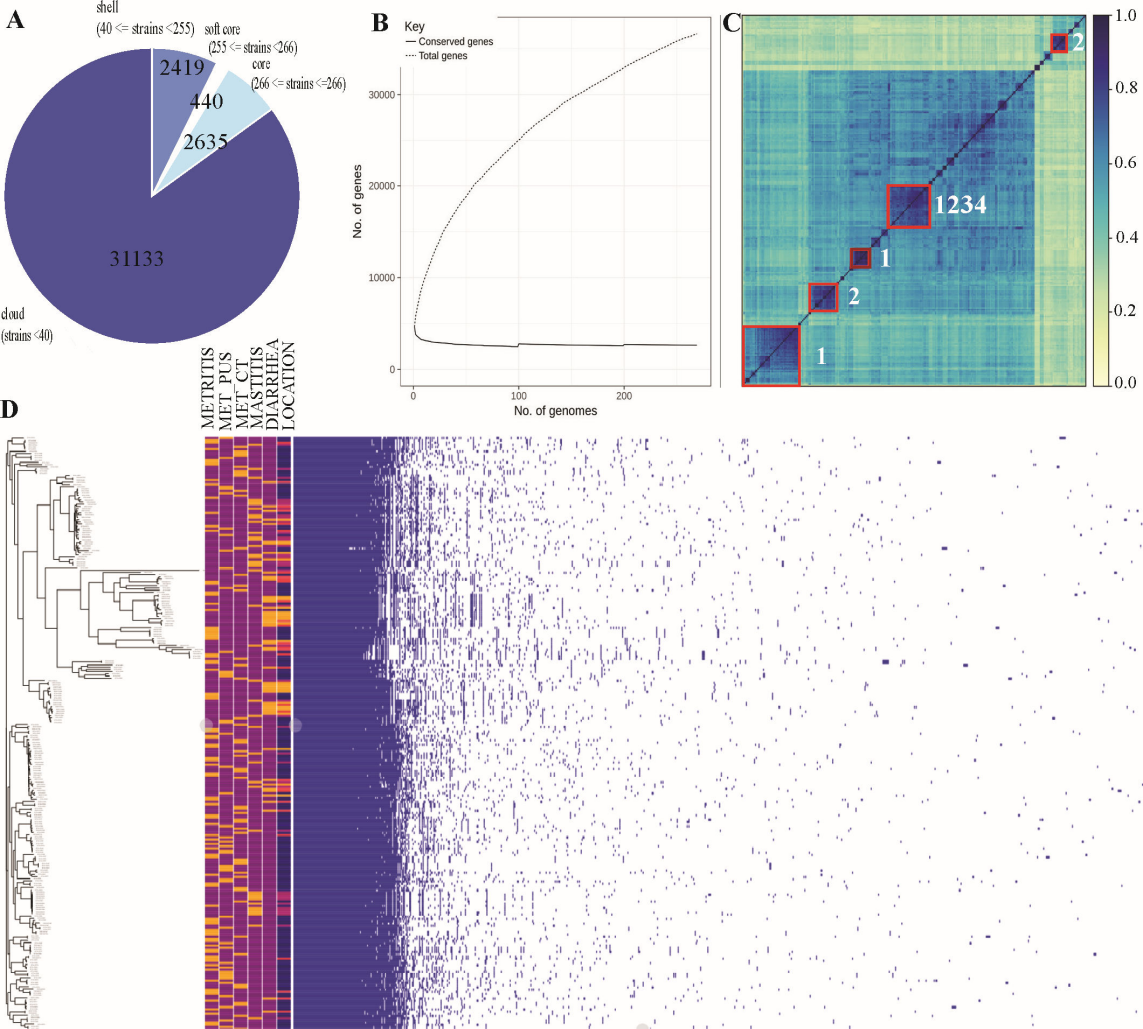
Pan-genome analyses of *E. coli* isolates. (**A**) Pie chart showing the proportion of repertoire genes in the core, soft-core, shell, and cloud of the pan-genome of the isolates (*n* = 268). (**B**) Pan-genome rarefaction curves showing the open pan-genome. (**C**) Whole-genome distance matrix depicting an all-against-all comparison of genome diversity for all isolates associated with disease phenotype. Disease phenotypes were labeled as number (1: mastitis, 2: diarrhea, 3: control metritis, and 4: metritis) (**D**) Gene presence-absence matrix of the gene distribution in each genome (orange: present, purple: absent), along with the metadata indicating the isolation source [metritis, metritis–purulent discharge (MET_PUS), control–metritis (MET_CT), mastitis, and diarrhea] and the geographic location (blue: United States, pink: Germany, orange: France, and yellow: Canada) of each genome.

The pan-genome from the 148 genomes isolated in this study was examined (Fig. S1) to find that the pan-genome was open. From this initial result, additional public WGS were used to determine the population pan-genome of 268 *E. coli* (148 from our study and 120 from NCBI). We found that this pan-genome was also open and contained 36,627 orthologous genes with 2,635 core genes and 33,992 accessory genes (softcore = 440, shell = 2,419, and cloud = 31,133) that represented the 7.2% and 92.8%, respectively (Fig. 1).

The population genome comparison of the genome distance among the 268 genome assemblies revealed two distinct groups with isolates from mastitis sources, two distinct groups with isolates from diarrhea sources, and a fifth distinct group with isolates from all clinical groups (Fig. 1C). Unexpectedly, isolates from uterine sources (metritis and no metritis) did not form an independent genomic cluster. These observations suggest that there are no specific genes in *E. coli* and, therefore, a specific *E. coli* pathotype that would increase an individual strain to more effectively result in infection of the uterus resulting in metritis in cattle.

The pan-genome analysis showed that the *E. coli* genomes from the uterus of cows with or without metritis were highly diverse and different from each other, again leaving the adaptation of *E. coli* to the uterus using genomic characterization unsupported. The Pan-GWAS analysis did not uncover any significant association between these genomes and virulence factors or antimicrobial resistance genes that allow the identification of specific or more virulent *E. coli* associated with metritis. The microbial pan-GWAS analysis determined several genomic features from the pan-genome associated with the clinical groups of diarrhea and mastitis (Table S3). Collectively, the genome diversity and pan-genome investigation did not support the theory that metritic *E. coli* are tissue adapted. With this assumption in mind, our findings do not exclude that generic *E. coli* can cause metritis in dairy cattle but instead that there is not a specific genomic marker associated with *E. coli* that was identified in cases of metritis in cattle. The large genomic diversity suggests that the disease complexity and progression are not associated with a single *E. coli* gene or genotype but instead with other factors, such as the host immune defense and uterine environmental factors or bacterial metabolic adaptation and physiological changes to stress response.

### Virulence factors and antimicrobial resistance gene analysis

**Fig 2:**
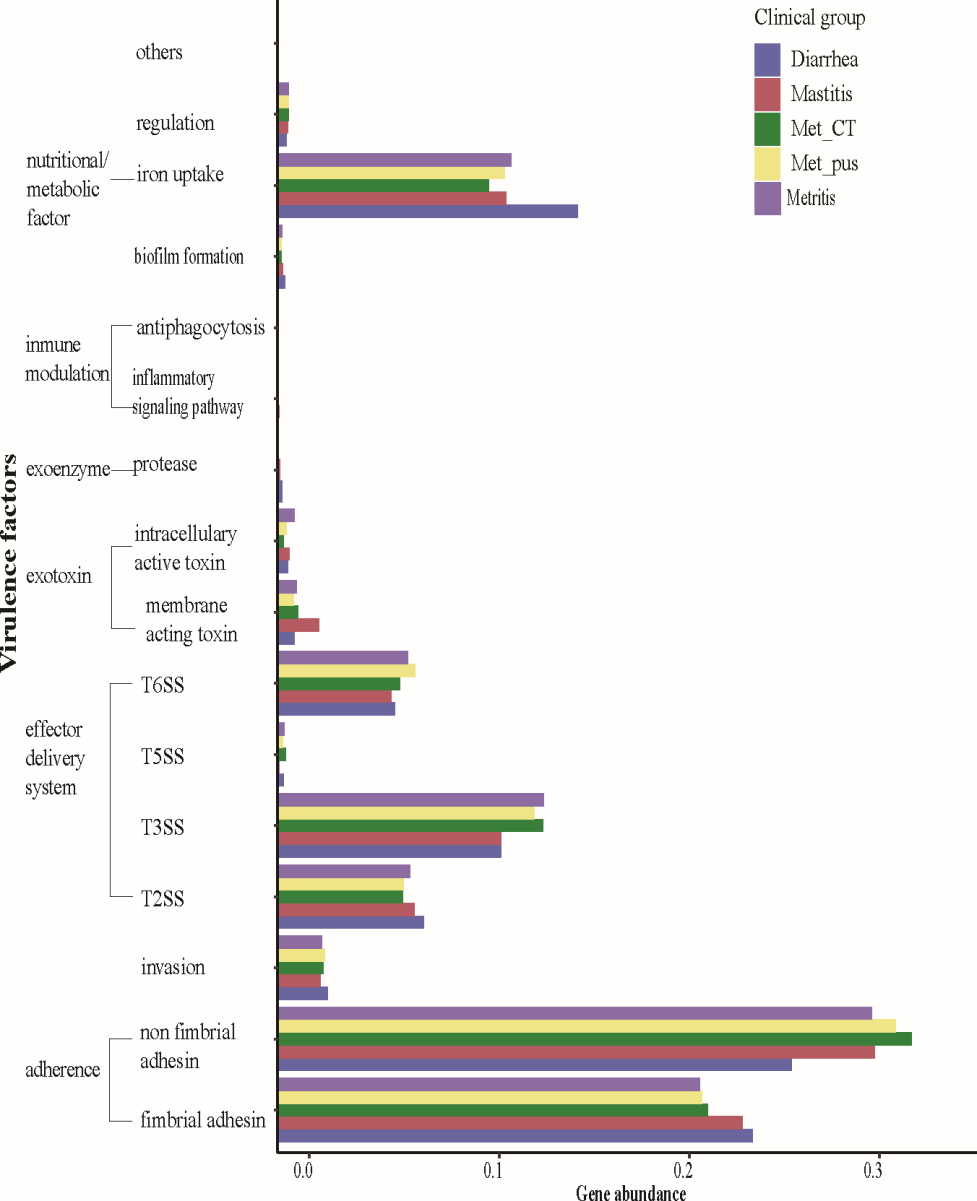
Virulence factor gene abundance as per health group and virulence factor category of 268 *E. coli* genomes from dairy cows classified in five different clinical groups.

**Fig 3:**
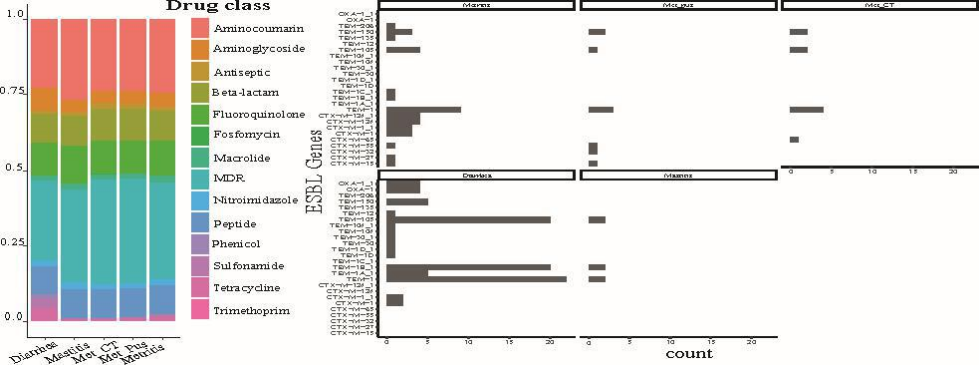
(**A**) Antimicrobial resistance gene abundance as per drug class between health groups of 268 *E. coli* genomes from dairy cows. (**B**) Prevalence of ESBL genes between health groups (*n* = 60).

There was a significantly higher gene abundance of the categories of iron uptake (%, *P* = 0.001), fimbrial adhesins (*P* < 0.001), and Type II secretion system (*P* = 0.001) in the genomes from diarrhea compared with the other clinical groups and a significantly lower gene abundance of Type III secretion system and non-fimbrial adhesins (*P* = 0.001). The genomes from mastitis had a significantly higher gene abundance of genes encoding for fimbrial (*P* = 0.01) and non-fimbrial adhesins (*P* = 0.01) compared with genomes from cows with or without metritis (Fig. 2). A gene previously believed to be associated with metritis (*fimH*) (25, 26) was identified in every genome in our study as well as in all genomes from the public domain, independently of the isolation source and, therefore, not associated with the clinical group (Tables S4 and S5). Three different *fimH* alleles were detected, without any statistical association among the allelic variation of the gene and any of the health groups. There was a significantly higher gene abundance for the drug classes of aminoglycosides (*P* < 0.001), sulfonamides (*P* = 0.001), and tetracyclines (*P* = 0.001) on the genomes from diarrhea when compared with the other clinical groups (Fig. 3A). The complete results from the permutational multivariate ANOVA (PERMANOVA) analysis are shown in Table S4.

**Fig 4:**
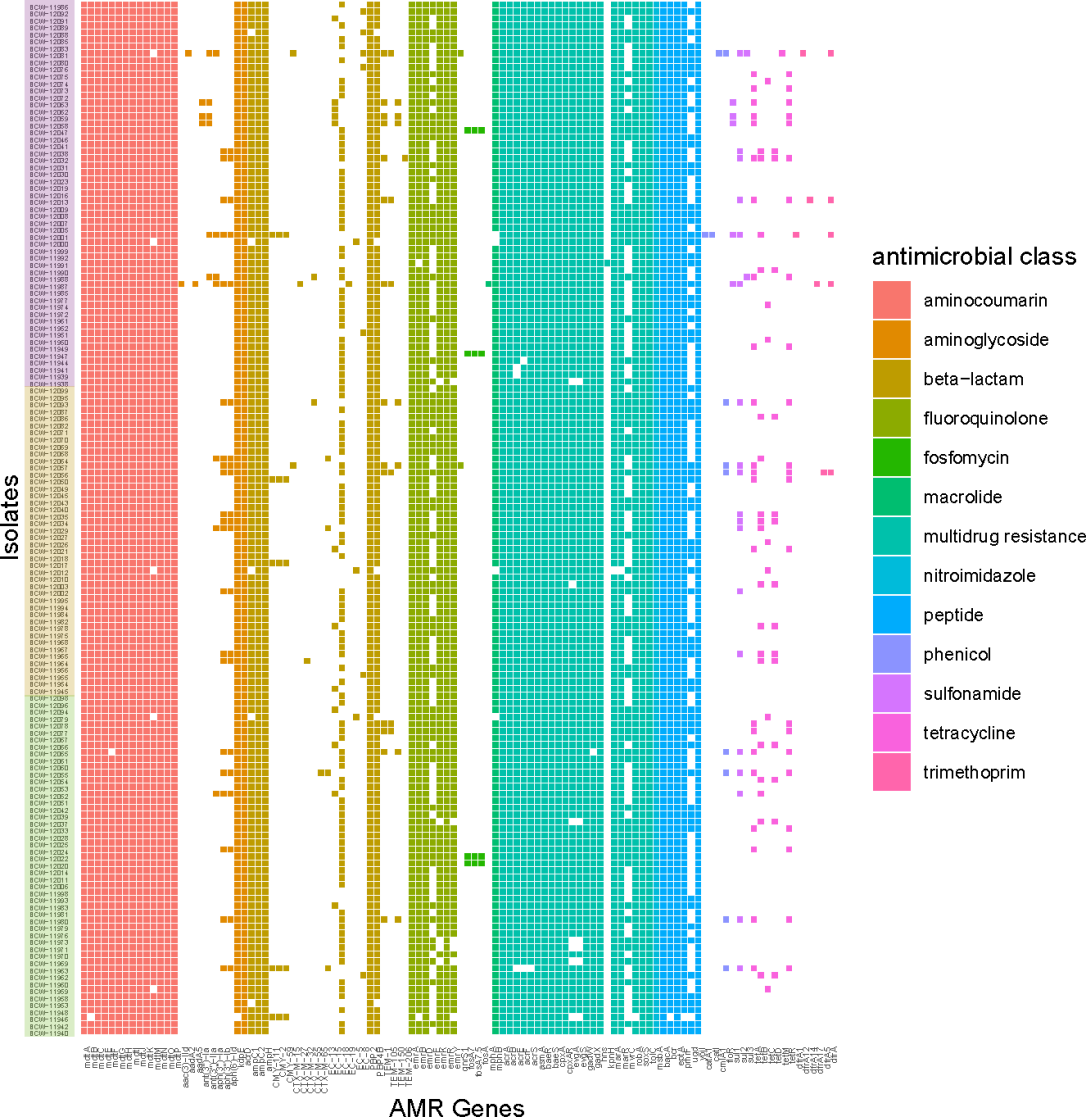
Presence or absence matrix of antimicrobial resistance genes by drug class and their corresponding health group (control: green, pus: orange, and metritis: purple) for 148 *E. coli* isolates from uterus of postpartum dairy cows.

Twenty unique ESBL genes and gene variants were identified in the genomes. The prevalence of genomes carrying ESBL genes was 24.2% (*n* = 60), with a median of three (range: 1–5) unique genes detected in a single genome (Fig. 3B). The genomes with a higher abundance of ESBL genes belonged to diarrhea and metritis clinical groups. At the univariate analysis, diarrhea genomes had higher odds of carrying *TEM-105* (OR: 6, CI: 3–14, *P* < 0.001), *TEM-1B_1* (OR: 15.1, CI: 5.7–54, *P* < 0.001), and *TEM-1* (OR: 3.5, CI: 1.9–6.8, *P* < 0.001), while metritis genomes had a higher odds of carrying *CTX-M-124* (OR: 18.9, CI: 2.3–623, *P* < 0.001) when compared with the other clinical groups. When comparing the 148 isolates from this study, no significant difference in the prevalence of antimicrobial resistance or virulence factor genes between the clinical groups and drug classes or virulence factor categories was observed. The genotypic profile of the 148 *E. coli* genomes from postpartum cows showed the presence of 504 unique virulence genes and 117 antimicrobial resistance genes as per drug class and resistance mechanism (Tables S5 and S6). Additionally, 116 unique serotypes were identified (Table S7). The most abundant resistance genes found at the drug class level were aminocoumarin (23.3%), followed by beta-lactams (12.3%) and fluoroquinolones (10.6%). A total of 33.8% of isolates were multidrug resistant. The most abundant virulence genes belong to the adherence class (55%), followed by the effector delivery system class (27%) (Fig. 4).

### Genotype–henotype agreement

The genotype-phenotype agreement found a variable accuracy in predicting the phenotype of the isolates having the genetic information (Table 2). Accuracy varied from 0.7% to 100%, with the lowest value being for enrofloxacin and gentamicin and the highest accuracy value for ampicillin. For most of the drugs, the high prevalence of false positives led to a low specificity for detecting genes corresponding to drugs for which they were phenotypically susceptible based on standard antimicrobial susceptibility testing methods (32).

**TABLE 2:** Comparison of phenotypic antimicrobial susceptibility testing and genome-derived resistance prediction *for E. coli* isolates (*n* = 148)

Antibiotic	AMR genes	Susceptible phenotype		Resistant phenotype		Accuracy	Sensitivity	Specificity
		Resistant genotype	Susceptible genotype	Resistant genotype	Susceptible genotype	(TP + TN)/total	TP/(TP + FN)	TN/(TN + FP)
		FP	TN	TP	FN			
Ampicillin	*ampC1* (*n* = 143), *ampC2* (*n* = 148), *ampH* (*n* = 148), *CMY-111* (*n* = 5), *CMY*-2 (*n* = 5), *CMY*-59(*n* = 5), *PBP2* (*n* = 148), *PBP4B* (*n* = 143), *CTX-M-15* (*n* = 2), *CTX-M-27* (*n* = 1), *TEM-1* (*n* = 14), *TEM-105* (*n* = 6), *TEM-150* (*n* = 7), *TEM-206* (*n* = 1)	0	0	148	0	1.00	1.00	0
Ceftiofur	*ampC1* (*n* = 143), *ampC2* (*n* = 148), *ampH* (*n* = 148), *CTX-M-15* (*n* = 2), *CTX-M-27* (*n* = 1), *CTX-M-32* (*n* = 1), *CTX-M-55* (*n* = 2), *CTX-M-65* (*n* = 1), *EC-13* (*n* = 4), *EC-15* (*n* = 12), *EC*-18 (*n* = 106), *EC-19* (*n* = 1), *EC*-5 (*n* = 3), *EC*-8 (*n* = 7), *TEM*-1 (*n* = 14), *TEM-105* (*n* = 6), *TEM-150* (*n* = 7), *TEM*-206 (*n* = 1)	130	0	18	0	0.12	1.00	0
Gentamicin	*aac*(3)-*Iid* (*n* = 1), *aac*(6′)-Iaa (*n* = 1), aac(6′)-Iy (*n* = 1), *aadA2* (*n* = 1), *aadA5* (*n* = 1), *ant*(3′′)-Ia (*n* = 3), *ant*(3′′)-IIa (*n* = 7), *aph*(3′′)-Ia (*n* = 3), *aph*(3′)-Ib (*n* = 21), *kdpE* (*n* = 147), *acrD* (*n* = 148)	147	0	1	0	0.007	1.00	0
Florfenicol	*catA1* (*n* = 1), *catI* (*n* = 1), *cmlA1* (*n* = 1), *floR* (*n* = 8)	2	132	7	7	0.93	0.50	0.98
Chlortetracycline	*tetA* (*n* = 22), *tetB* (*n* = 18), *tetC* (*n* = 7), *tetD* (*n* = 14), *tetM* (*n* = 1), *tetR* (*n* = 22)	7	97	40	4	0.92	0.90	0.93
Oxytetracycline		3	93	44	8	0.92	0.84	0.96
Enrofloxacin	*emrA* (*n* = 148), *emrB* (*n* = 146), *emrD* (*n* = 148), *emrE* (*n* = 65), *emrK* (*n* = 143), *emrR* (*n* = 148), *emrY* (*n* = 142), *qnrS1* (*n* = 2)	147	0	1	0	0.007	1.00	0
Danofloxacin		141	0	7	0	0.04	1.00	0

## DISCUSSION

This study describes the first population-scale (*n* = 148) genomic comparison of *E. coli* genomes collected from postpartum dairy cows from multiple farms and generated data that do not support previously recognized concepts of tissue adaptation and specific gene virulence factors in *E. coli* isolated from cows with metritis having unique virulence factors when compared with cows without metritis. This observation aligns with that of other organisms that have complex disease progress such as *Helicobacter pylori* where the genotype evolves during the disease (33). Furthermore, sequencing population WGS revealed that the pan-genome of uterine *E. coli* did not differentiate among the clinical groups and that there was no evidence of a specific genotype associated with metritis or uterine isolation source; therefore, the traditional EnPEC (34) pathotype was not evident in the isolates from this study and it did not emerge with additional public WGS to increase the analytical power of the analysis. An explanation for these findings is that the well-recognized plasticity and genetic diversity of *E. coli* contribute to its ability to adapt to different ecological niches (28, 35, 36). A broader comparison of our metritis genomes to those from other metritis studies and isolated from animals with diarrhea and mastitis further supported the hypothesis of a non-unique genotype in the metritis isolates, revealing instead that multiple genotypes comprise the pathotype previously referred to as EnPEC (Fig. 2). Unlike other ruminant pathogens, such as *Campylobacter jejuni* which causes abortion in goats and sheep, where a single gene allele of *porA* was found to be selected over 35 years and six geographic locations to induce disease (37), we did not find any evidence of the selection or adaptation to the uterus. Our findings highlight the discriminatory power of WGS, which has been similarly used under other disease conditions to outline incongruencies when compared with more typical serotyping classification methods on genomes from avian pathogenic *E. coli*, as an example (38).

Our study found significant differences in the prevalence of virulence genes in *E. coli* from the mastitis and diarrhea groups, but not on the genomes of uterine origin. Previously conducted studies have evaluated *E. coli* virulence factors from uterine samples on postpartum dairy cows yielding contradictory results regarding their association with metritis. Most of these studies have used PCR as the detection method limiting the results to the preselected genes to be screened. Silva et al. (39) characterized the genomic profiles of *E. coli* recovered from postpartum dairy cows; in their study, 72 isolates from healthy (*n* = 35) and metritic (*n* = 37) cows within the first 42 days in milk (DIM) were screened for virulence genes using PCR, and from the 15 screened virulence genes, only 6 were identified in the samples and none of them were associated with uterine health status (39). Similarly, Sheldon et al. (34) did not find an association between uterine disease and 17 different virulence genes screened in 114 *E. coli* isolates recovered from cows that were up to 4 weeks postpartum; all isolates evaluated in their study did not possess any of the 16 examined virulence genes, and only a gene associated with iron uptake (*fyuA*) was identified in isolates from cows with uterine infection while the gene was not identified in any of the isolates retrieved from healthy cows (34). In contrast to these studies, Bicalho et al. (26) evaluated virulence factors from *E. coli* isolated from 125 dairy cows within 3 to 7 DIM using PCR; for their study, *E. coli* isolates were screened for 32 different VF, and a significant association between six VF (*fimH*, *astA*, *cdt*, *kpsMIII*, *ibeA*, and *hlyA*) in *E. coli* and metritis was observed when compared with culture-negative cows (26). A limitation of this study arises from the study design and data analysis, where the risk of specific VFs (e.g., *Fim*H) for causing metritis was compared with that of cows with a negative intrauterine *E. coli* culture swab result (cows with no *E. coli* isolated from the sample), instead of a culture-positive sample (cows with *E. coli* without the specific VF isolated). Under these circumstances, the presence or not of *E. coli* is the only factor that can be compared when evaluating the risk for metritis. To evaluate the effect of specific VF, an appropriate reference group with an *E. coli* culture positive and VF negative is needed to compare an *E. coli* culture-positive VF present and the risk of metritis when comparing both groups, as has been done in our study.

A similar study conducted in 2012 by the same research group (25) extracted DNA directly from intrauterine swabs to evaluate the association between *E. coli* carrying only the virulence gene *fimH* and metritis using PCR. They found *E. coli* isolates carrying *fimH* during all sampling points through the study (1–3, 8–10, and 36–36 DIM) with a higher percentage during day 8, when compared with cows with PCR-negative test results for *E. coli* isolates carrying the *fimH* gene. The study also found an increased odds ratio (OR 4.7, *P* < 0.01) for metritis in cows when *E. coli* carrying *fimH* was isolated within the 1–3 DIM. A limitation of the Bicalho et al. study (25) was similar to the one outlined above for their 2010 study, where a lack of an appropriate control group (*E. coli* positive by PCR but not carrying *fim*H or other VF of interest) limits any conclusions for the association of *fim*H as the causative risk factor for metritis, and the result probably should be restricted to present or not of *E. coli* with positive test results as determined by PCR, with not conclusion alluding an association of specific VF with metritis.

A recent study by Kassé et al. (24) evaluated 371 cows within 3–20 DIM, for 40 different virulence genes using PCR (24), and identified 32 virulence genes among the isolates, with two genes being associated with metritis (*hra1* and *kpsMTII*). In contrast with the study by Bicalho et al. (26), the Kassé et al. (24) study found that *fimH* was the most abundant gene found (89%) in both metritis and healthy animals, and therefore, it was not considered a factor associated with uterine disease (24). Like the Kassé study (24), our study found *fimH* was not associated with metritis; likewise, our results go further evaluating 504 VF genes, without any specific VF associated with metritis.

FimH encodes an adhesive subunit protein of type 1 fimbriae responsible for D-mannose-sensitive adhesion, an important determinant for adhesion and subsequent colonization of mucosal surfaces (40–42). More than 85% of *E. coli* (commensal and pathogenic) expresses type 1 fimbriae (43–45). However, FimH has been recognized as an important VF for invasion of the urinary epithelium in urinary tract infections, as well as of intestinal epithelial in colitis (46) and IBD, and associated with adherence, invasion, and inflammatory response in Crohn’s disease (47). Together, our findings indicate that *fim*H is a core gene that will be found in all *E. coli* isolates associated with cattle, rather than a VF that is causal to the development of metritis in dairy cows. These observations highlight the need to use appropriate control groups and population genomics to understand gene distribution in highly mutable pathogens.

Multiple factors could have influenced the difference in results between the three studies outlined above using PCR for gene detection, including study design and inclusion of control groups as previously mentioned. A more in-depth evaluation of the inconsistency of detection for important virulence genes of bacteria such as Shiga toxin-producing *E. coli* has been addressed before (48–50). Furthermore, WGS evaluation provides a much more robust method with greater comprehensive and discriminatory power when compared with primer-dependent methods such as PCR, which has various limitations that can be overcome by new sequencing technologies (51). Allele variations in a gene can result in a gene not being detected using PCR approaches, whereas WGS would not have this limitation. Because of these advantages, WGS is considered a more effective and reliable testing approach when compared with PCR approaches (51, 52). Our study represented one of the first studies using a deep-sequencing approach from a broad source of intrauterine *E. coli* to evaluate factors associated with postpartum metritis in dairy cattle (27, 53).

Regarding antimicrobial resistance genes, our study found that the most abundant genes were found to confer resistance to aminoglycosides, tetracycline, and beta-lactams. Similarly, Jeamsripong et al. (54) also found the highest prevalence of resistance in fecal *E. coli* from dairy cattle was to tetracycline (27.5%), sulphonamide (22.5%), and aminoglycoside (20.0%). A multicentric study in Europe evaluated the genomic profile of 150 fecal *E. coli* isolates from veal calves and found that the most common AMR by drug class were against tetracycline (67%), aminoglycoside (61%), sulphonamide (58%), and beta-lactam (49%). Likewise, the most prevalent genes associated with resistance were *tetA* (56%), *aph(6)-Id* (43%), *sul2* (40%), *aadA1* (39%), and *TEM-1B* (30%) (55). Broad similarities found between our metritis genomes when compared with fecal samples reflect the findings also observed when comparing the abundance of antimicrobial resistance genes between intrauterine *E. coli* from our study and those from mastitis and diarrhea cases.

To our knowledge, only one previous study aimed to characterize the AMR genotype of *E. coli* from postpartum dairy cattle (27). This study found 71 different AMR genes in 13 *CTX-M*-positive isolates and 1 non-ESBL isolate from 4 cows with metritis. However, a small sample size, a low threshold for gene identification (>70% similarity), and a lack of a control group comparing *E. coli* from cows without metritis limit the generalization of their findings. Our study overcame this limitation by including *E. coli* from control animals and not utilizing a selective media only to characterize ESBL-positive strains, therefore representing a broader outlook of intrauterine *E. coli* from dairy cows.

The emergence and dissemination of ESBL genes have become a major concern in public health (29, 30). Although there is a knowledge gap about the directionality in the dissemination of AMR bacteria and their resistant determinants between food animals and humans (56), ESBL represents a challenge for the successful treatment of infectious diseases (57, 58). Our results showed that *TEM-1*, *TEM-105*, and *CTX-M-124* were the most abundant ESBL genes and were associated with diarrhea (*TEM-1* and *105*) or metritis (*CTX-M-124*). *TEM-1* is one of the most widely studied ESBL genes and has been found in *E. coli* distributed globally (59). TEM enzymes represent an important concern to veterinary medicine, for their effect on penicillins and early-generation cephalosporins, which are commonly used in lactating cattle (31). *CTX-M-124*, a *CTX-M-2* variant (60), was first reported in wild birds (61) and has also been described in corvids (62) and dogs and cats admitted to a veterinary teaching hospital (63). *CTX-M* enzyme family confers resistance to cefotaxime (64), a third-generation cephalosporin closely related to the primary antimicrobial use for metritis treatment (ceftiofur) (1, 65). Further research is needed to understand the role of this resistance gene and the implication of treatment success.

Our findings demonstrated a moderate overall accuracy for whole-genome sequencing to predict phenotypic resistance, which considerably varied depending on the specific antimicrobial tested. Previous studies have evaluated the correlation between WGS AMR genes and antimicrobial susceptibility, and the accuracy of predicting antimicrobial susceptibility of isolates has also reported a highly variable correlation dependent on the antimicrobial drug (66–69). A study evaluating the AMR profile of 150 fecal *E. coli* isolates from beef cattle reported a positive genotype and phenotype correlation (*r* ≥ 0.85; *P* < 0.05) of the isolates. The study also found that most of the discordant results came from isolates harboring genes responsible for aminoglycoside and sulfonamide resistance (70). Similarly, reference 54 described the antimicrobial resistance profiles of 40 fecal *E. coli* from dairy cattle at different production stages on a commercial farm. Results from this study found a high agreement (85.71%–97.50%) between the resistance phenotype and the presence of resistance genes. However, kappa statistics varied greatly between antimicrobials, showing a weak level of agreement between aminoglycoside resistance genes and streptomycin (39.5%) and trimethoprim and trimethoprim–sulfamethoxazole (48.9%) resistance and a moderate agreement for beta-lactamases ampicillin (78.7%) and tetracyclines (60.8%). Genotype–phenotype correlation variation among studies might be influenced by several factors such as small sample size, databases included for AMR gene identification, thresholds used for gene identity and coverage, and MIC values used for susceptibility classification of isolates. Results of genotypes in the current work and phenotypes in our previous work (32) on the same bacterial isolates allowed us to better understand the resistance of uterine *E. coli* on dairy farms.

Different factors involving the host genetics and immune response, the epithelial barrier disruption at calving, and the uterine microbiome warrant further research to better understand the pathogenesis of the disease. Changes in microbiota after calving and the reduced diversity have been described before in metritic cows compared with healthy cows (11, 16, 17). Microbiome studies have found that the dynamics of uterine microbiota differs between cows with metritis and control cows and have attributed the disease as a consequence of uterine dysbiosis (71–73). This dysbiosis favored the overgrowth of facultative anaerobic bacteria such as *Fusobacterium* spp. and *E. coli*, which could explain the higher prevalence of *E. coli* isolated from cows with metritis than from control cows or cows with purulent vaginal discharge found in the present study.

*E. coli* has been identified as the causal agent of metritis using culture-based methods, and the disease has been successfully replicated in *in vivo* models using *E. coli* or in combination with other bacteria such as *T. pyogenes* (19, 20, 74). Microbiome studies have found a variable association between *E. coli* and metritis when compared with control cows (72, 73). Differences in disease development among these groups may benefit from a better understanding of the evolution of pathogenic *E. coli*. The high genetic plasticity along with the horizontal gene transfer of pathogenicity islands favor the rapid adaptation of *E. coli* to new ecological niches (75, 76), allowing commensal strains to have pathogenic potential or act as genetic reservoirs for virulence factors and AMR genes (75). Finally, understanding the host factors relevant to the development of the disease such as the metabolic status of the cows at calving and the negative effect on their immune system, as well as the interaction of the host immune response with pathogenic bacteria, may help understand the differences in prevalence and disease severity among animals. The role of *E. coli* has been discussed in human diseases in which pathology is not completely understood yet either, like in ulcerative colitis, and the bacteria’s ability to cause disease depends on the host immune status, the intestinal microbiota, and the intestinal immune response (77). A better understanding of the interaction of *E. coli* in the uterine immune response in postpartum cows may help elucidate the differences in disease development when the bacteria is present.

### Conclusion

An *E. coli*-specific genotype was not found to be associated with metritis in this study. This undermines the concept that *E. coli* are tissue adapted as was found with other dairy cattle *E. coli-*related diseases. In contrast, a high genetic diversity among the isolates from uterine sources (metritis, PUS, and control) is in conflict with the current classification for uterine-specific *E. coli* niche adaptation theory. A virulence factor previously associated with *E. coli* in metritis (*fim*H) was equally prevalent between all isolation sources and found to be a core gene in the population pan-genome comparison and was not specifically associated with the development of metritis. Although three different *fimH* alleles were identified across the *E. coli* genomes, there was not any association between allele variation and metritis. ESBL genes were more abundant in genomes from diarrhea and metritis clinical groups. Genomes from the metritis clinical group had higher odds of carrying the *CTX-M-124* ESBL gene, while genomes from the diarrhea group had higher odds of carrying *TEM-*105, *TEM-1B_1*, and *TEM-1* ESBL genes. Our findings generated novel data that provide significant advancement to the field, for redefining the traditional definition of uterine-specific *E. coli* and highlighting the need for continuous revision of previous concepts of metritis pathogenesis using new deep-sequencing technologies. Further research that investigates the uterine microbiome in cows with and without metritis, as well as the role of the host immune response in disease development and postpartum involution, may help better understand metritis pathophysiology and tailor interventions for disease diagnosis and successful treatment.

## MATERIALS AND METHODS

### Sampling and study design

All procedures were approved by The University of California Institutional Animal Care and Use Committee (no. 20620). The study was conducted between September 2018 and November 2019.

**Fig 5:**
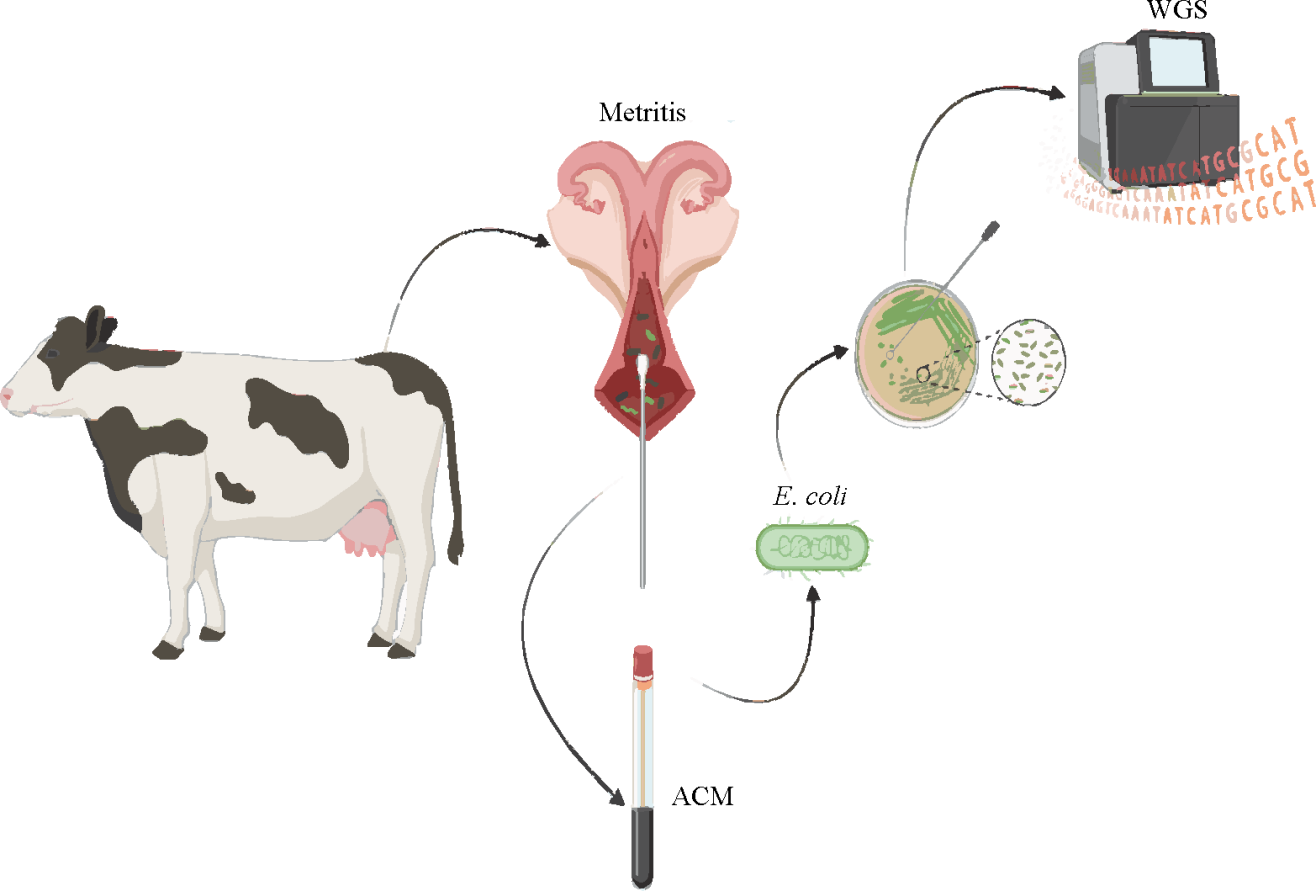
Graphical summary depicting the study design and methodology used to isolate and sequence intrauterine *E. coli* (*n* = 163) from cows with and without metritis.

Bacterial isolates (*n* = 163) were collected as part of a larger cross-sectional study that collected uterine swabs from postpartum cows between 3 and 21 days in milk using a convenience sample of 25 commercial dairy farms from the Sacramento and San Joaquin Valleys in California (32). Three clinical groups were defined based on VD characteristics as metritis discharge: watery, reddish, or brownish, and fetid, purulent discharge: non-fetid purulent or mucopurulent vaginal discharge, and normal discharge: clear lochia, clear mucus, or no vaginal discharge (Fig. 5).

### DNA extraction, library preparation, and whole-genome sequencing

Frozen bacterial stocks were used to inoculate a CHROMagar- *E. coli* selective plate (CHROMagar Microbiology, Paris, France) and were incubated aerobically at 37°C for 24 hours. A single, well-isolated colony was subcultured in 1.5 mL of sterile Luria–Bertani broth (Difco; Becton, Dickinson, and Company, Sparks, MD, USA) at 37°C for 24 hours. Culture tubes were centrifuged at 10,000 × *g* for 3 min at room temperature (15°C–25°C), and the pellet was then resuspended in 180 µL Buffer ATL.

DNA was extracted using DNeasy Blood & Tissue (QIAGEN, Hilden, Germany), following the manufacturer’s instructions, and DNA concentration was measured at 260 nm using a NanoDrop One^c^ Microvolume UV-VIS spectrophotometer (Thermo Fisher Scientific Inc., Waltham, MA, USA). Additionally, for samples with a concentration < 50 ng/µL or A260/260 < 2.0, DNA was purified using Genomic DNA Clean & Concentrator-25 (Zymo Research Corp., Irvine, CA, USA).

DNA library preparation was conducted in the laboratory of Dr. Bart Weimer (UC Davis). DNA was analyzed on the Agilent 2200 TapeStation System using the Genomic DNA ScreenTape assay for the integrity of gDNA. Libraries were constructed using the KAPA HyperPlus Library Preparation Kit (Roche, Indianapolis, IN, USA), as previously described (78, 79). Whole genome sequencing was done using the Illumina HiSeq X platform with PE150 (Illumina Inc., San Diego, CA, USA) (80).

### Sequence assembly, annotation, and pan-genome analyses

The genome sequences were assessed for sequencing depth (>20× estimate), checked for quality using FastQC (v0.11.9) (81), and trimmed using Trimmomatic (v0.39) (82). Sequences were assembled using Shovil (v1.0.4) (83), checked for quality, size (4.5–6.5Mbp genome), completeness (>95% estimate), and contamination (<10% estimate) using CheckM (84), and assessed for approximate genera and species and further identity test for possible contamination using Kraken (85–90). Sixteen sequences that did not meet quality criteria were removed from downstream analysis.

The comparative WGS analyses were extended to include 130 public genomes of *Escherichia coli* of clinical importance from cows with diarrhea (*n* = 50, BioProject: PRJEB32666), cows with clinical mastitis (*n* = 50, BioProject: PRJNA612640), and cows with metritis (*n* = 30, BioProject: PRJNA298331); these genomes were combined with the 148 *E. coli* from our study to examine their genome relatedness, following the same procedures described above. Genome distance metrics were determined using all-by-all whole genome comparisons using Sourmash (v3.2.3). The genome distance metric was calculated using genome-wide k-mer signatures, using a k-mer size of 31 with sketch sizes scaled to 100,000/megabase. Pairwise comparisons were visualized as an all-against-all heatmap (91).

Core and accessory genes were annotated using Prokka (version v.1.14.6) (92). Pan-genome comparisons, identifying gene clusters and the core genes, were conducted using Roary (3.12.0) using 95% amino acid sequence identity (93) and visualized using Phandango (94) with the associated metadata. Gene associations, metadata, and phenotypes were associated using Scoary 1.6.12 (95). Finally, variant calling was done with the reference sequence *E. coli* ECC-1470 with Snippy (version v. 4.6.0) with default settings (96).

### Genomic assessment of virulence factors and antibiotic resistance genes

Virulence factors and antimicrobial resistance genes were analyzed in every genome using ABRicate (version 1.0.0) (97). Virulence factor genes were screened against the virulence factor database (VFDB), and ecoli_VF. Antibiotic resistance genes were screened against the Comprehensive Antibiotic Resistance Database (CARD), the Antibiotic Resistance Gene-Annotation (ARG-ANNOT), MEGARes, ResFinder, and the National Database of Antibiotic-Resistant Organisms (NDARO). The AMR genes were classified according to resistance mechanisms and drug class using the CARD database and manual curation (98). The VF genes were classified according to the classification scheme proposed by the VFDB (99). Both AMR and VF determinants were retained for analysis if they fit the minimum criteria of 90% identity and coverage. Serotyping was done using the ECOH database (100).

### Genotype–phenotype correlation for antimicrobial resistance

Genotype–phenotype correlation (GPC) analysis was performed using broth microdilution antimicrobial susceptibility test results (32) in combination with the WGS-informed AMR analysis. These assays were compared with the occurrence of known AMR genes with resistance to the respective drug. False-positive, false-negative, sensitivity, specificity, and accuracy of the GPC were calculated as previously described (66).

### Statistical analysis

Data analysis was conducted using RStudio (version 4.1.2). Microbial pan-GWAS analysis to identify significant gene associations was conducted using Scoary, and *P* values were adjusted using Bonferroni correction (95). Descriptive statistics were used to examine the distribution of AMR and VF genes in *E. coli* between the clinical groups. To investigate differences between the drug classes or the virulence factor category among the clinical groups (control, pus, metritis, diarrhea, and mastitis), PERMANOVA was conducted using the vegan package (101). Post hoc pairwise comparisons with Bonferroni correction were calculated for the categories with significant differences. Univariate analyses were conducted to evaluate the association between the prevalence of ESBL genes and the clinical group. Finally, to assess the agreement between *E. coli* isolates having a specific genotype and the corresponding resistant phenotype, the accuracy, sensitivity, and specificity were calculated. Statistical significance was set for all tests at *P* ≤ 0.05.

## Data Availability

All raw genome sequences generated in this study are available at the 100K Pathogen Genome Project BioProject (NCBI PRJNA186441) under BioProject accession number PRJNA1095494.

## SUPPLEMENTAL MATERIAL

Supplemental Tables mbio.01027-24-s0001.xlsx WGS tables.

ASM does not own the copyrights to Supplemental Material that may be linked to, or accessed through, an article. The authors have granted ASM a non-exclusive, world-wide license to publish the Supplemental Material files. Please contact the corresponding author directly for reuse.
